# Prevention of Infant Mortality

**Published:** 1900-09

**Authors:** 


### Abstracts.

### PREVENTION OF INFANT MORTALITY.

ERNEST WENDE, Health Commissioner of Buffalo (*Pediatrics*), in his annual report insists that it is necessary for state and municipality to have supervision over the milk supply.

1. Continuation of the license system with penalties, together with stringent ordinances covering quality, adulteration, sanitary care, and relation to contagious disease contamination.

2. Milk should comply with the state standard as determined by lactometer tests, periodically instituted both in milk houses and on wagons.

3. Sanitary nonabsorbent milk-rooms of determined size, air-space, ventilation, and light; with specially constructed, indirect-plumbed cooling and storage boxes not communicating with sleeping rooms, privies, stables, or other influencing rooms.

4. Wagons properly lettered and numbered, with protection from summer heat.

5. Stringent prohibition regarding intercourse with houses containing contagious diseases, particularly relating to the exchange of milk bottles and the prohibition of refilling milk jars en route. The constant surveillance of contagious disease in relation to milk routes by means of "tell-tale register."

6. Obligatory cleansing of milk utensils by a uniform method, and of cans before returning to the dairy.

7. Special supervision of and discouragement, if not abolishment of the sale of milk in groceries and similar stores where the business is surbordinated to other interests and consequently lacks protective facilities.

United States and city act in unison in this, where interests are identical, the adoption of arbitrary interdiction or destruction at the city line of all milk coming from unsatisfactory dairies.

Systematic reports, and mutual warnings and notification of imperiling conditions. — *Medical and Surgical Monitor*, August, 1900.

